# Effect of Fermentation on Nutritional Quality, Growth and Hematological Parameters of Rats Fed Sorghum‐Soybean‐Orange flesh Sweet Potato Complementary Diet

**DOI:** 10.1002/fsn3.2013

**Published:** 2020-12-08

**Authors:** Kikelomo P. Adejuwon, Oluwatooyin F. Osundahunsi, Stephen A. Akinola, Matthew O. Oluwamukomi, Mulunda Mwanza

**Affiliations:** ^1^ Department of Nutrition and Health Promotion Ondo State Primary Healthcare Development Agency Akure Nigeria; ^2^ Department of Food Science and Technology Federal University of Technology Akure Ondo State Nigeria; ^3^ Department of Microbiology Food Security and Safety Niche Faculty of Natural and Agricultural Sciences North West University Mmabatho South Africa; ^4^ Center for Animal Health Studies Food Security and Safety Niche Faculty of Natural and Agricultural Sciences North West University Mmabatho South Africa

**Keywords:** complementary food, fermentation, orange‐fleshed sweet potato, protein, sorghum, soybean

## Abstract

The protein quality of complementary foods developed from fermented and unfermented sorghum, soybeans, and orange‐fleshed sweet potato (OFSP) flour blends was evaluated using rat model. The test diet was as follows: UF2: unfermented sorghum (56%), soybean (17%), and OFSP (27%); UF3: unfermented sorghum (59%), soybean (31%), and OFSP (10%); F2: fermented sorghum (56%), soybean (17%), and OFSP (27%); and F3: fermented sorghum (59%), soybean (31%), and OFSP (10%), while cerelac served as positive control, corn starch (basal diet), and *ogi* (negative control). Forty‐nine Wistar albino rats were grouped and fed with diets for 28 days. The growth, hematological, serum parameters of animals, protein quality, and proximate composition of developed diet were determined. Fermentation significantly improved the protein content and nutritional indices of experimental animals. Moisture content ranged from 2.5% to 9.24%, protein (7.09%–25.29%), ash (1.09%–3.71%), fat (10.28%–15.24%), and fiber (0.85%–3.17%). The biological values (BV) ranged from 75.11% to 78.44%. The weight gained in rat fed the formulated diet ranged from 46.0 g to 77.3 g and was highest in F3. The packed cell volume (PCV), hemoglobin concentration (HBC), red blood cell (RBC), and lymphocytes were highest in F3. Urea nitrogen and creatinine of the rats fed with formulated diets ranged from 3.58 to 15.32 mg/dl and 1.56 to 6.15 mg/dl, respectively. Sample F3 is a protein‐rich complementary food that is comparable to ogi and suitable to manage malnutrition and support growth in children. However, clinical trials on the formulated diet are needed to further substantiate its nutritional potentials.

## Graphical abstract

A complementary diet can be successfully developed from fermented sorghum–soybean–orange‐fleshed sweet potato. Fermentation improved the protein and nutritional quality of developed weaning diet and health parameters of rats. Samples F3 supports growth and could be used as a complementary food for infants and as substitute to local complementary food ogi.

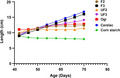

## 1 INTRODUCTION

Generally, malnutrition refers to both under and over nutrition. It is a condition that develops from an inadequate supply of the body with proper amount of protein, energy (calories), vitamins, and other nutrients to maintain healthy tissues and organ function. Malnutrition among children is one of the most important causes of morbidity and mortality in the world, particularly in developing countries (Thapar & Sanderson, 2004). It is the most important risk factor for disease in children causing about 300,000 deaths annually (Alemu et al., 2005). Malnutrition has been directly or indirectly linked to more than half of deaths cases in children (UNICEF, 2017). According to the reports of Tariku et al. (2017), about 21.9% of children under the age of five are stunted, while 13.4% are underweight, and 7.3% are wasting. Also, the World Health Organization had estimated that about 5.4 million under‐five age children die each year with 2.7 million deaths in Africa including Nigeria (Hug et al., 2017).

Several attempts to solve the challenges of malnutrition among children includes the development of complementary diets; however, in the developing countries this have experienced a setback due to poverty; hence, researches have focused on the development of complementary foods from blends of local staples. Oyarekua & Adeyeye (2009) reported the nutritional quality of developed weaning food from strategically combined fermented sorghum, maize, and cowpea flour. Similarly, Adegbanke et al. (2018) reported the development of complementary diet from blends of germinated sorghum, peanuts, and Irish potato flour. Among others are fermented sorghum–ginger–walnut (Adebayo‐Oyetoro et al., 2012), Sorghum–soybean–sesame flour (Makinde & Lapido, 2012), and amaranth–sorghum diets (Okoth et al., 2017). This study further investigated the use of locally available staples in the development of a complementary food that could help to manage malnutrition in children especially those in developing countries.

In Nigeria, the first‐line complementary food is cereal gruel, which is low in protein and nutrient density (Ijarotimi, 2013; Issaka et al., 2015). Traditional foods often fail to meet the nutritional needs of infants due to poor nutritive values (Fernandez et al., 2002; Issaka et al., 2015). Most plant‐based complementary foods are not able to adequately supply the protein and certain micronutrient requirements for a growing child (Issaka et al., 2015). Similarly, cereal‐based complementary foods have been implicated in the etiology of protein energy malnutrition especially in communities where it is solely used as complementary food (Appoh & Krekling, 2005; Issaka et al., 2015). Several studies have reported that traditional complementary food (ogi) is characterized with low lysine and tryptophan, low energy density, and bulky (Issaka et al., 2015; Kikafunda et al., 2006; Osundahunsi & Aworh, 2002). In order to improve the protein quality of cereal‐based complementary diets, combination of varieties of food materials such as staple starchy roots, legumes, and plant proteins such as soybeans and groundnut is considered appropriate for developing weaning foods (Issaka et al., 2015; Ugwu, 2009).

Orange‐fleshed sweet potato (*Ipomea batatas* [L.] *Lam*.) is a dicotyledons plant from the family Convolvulaceae that grows in tropical and subtropical areas (Dasgupta et al., 2008). Over 7 million tons of sweet potatoes are produced in Africa most of which is produced from the East and Southern Africa region (Olapade & Ogunade, 2014). Orange‐fleshed sweet potatoes (OFSP) is a good source of energy (293–460 kJ/100 g) and vitamin A that is better absorbed than others leaves and vegetables (Amajor et al., 2014; Bengtsson et al., 2009). Soybean (*Glycine max*) is a legume native to East Asia and widely grown for its edible bean (Manu et al., 2020). It is the fourth most important crop that provides oil and protein (Srinivasan et al., 2017). According to El Nehir and Simsek (2012), soybean have relatively high proteins (40%–50%), fat (20%), and low in carbohydrates (35%). Sorghum (*Sorghum bicolour*) is the fifth most important cereal crop (Breeze, 2018) and contributes significantly to the energy requirements of people, especially those in Africa and Asia (Elkhier & Hamid, 2008). Tannins found in sorghum binds protein, carbohydrates, and minerals, thereby affecting the nutritional and functional value of the bound constituents (Aluge et al., 2016). The biological availability of minerals may be impaired by phytates found in sorghum (Choi & Rhee, 2006; Rajkumar et al., 2012) especially when they form insoluble precipitates thereby preventing absorption. More effective methods that have been used to improve bioavailability of nutrients in foods include the following: soaking, fermentation, heating, and sprouting (Isanga & Zhang, 2008; Rajkumar et al., 2012). Fermented foods play an important role in providing food security, enhancing livelihoods, and improved nutrition and social well‐being of the people (Adesulu & Awojobi, 2014). Fermentation leads to improved food preservation (Ross et al., 2002), food quality, and increase range of edible food products. An enhancement of the nutritive value by an increase level of essential nutrients or reduced levels of toxicants in food remains a benefit of fermentation process (Evans et al., 2013). Hence, the protein quality of fermented and unfermented sorghum, soybeans, and orange‐fleshed sweet potato blends was investigated through a rat model. The aim of this study was to investigate the protein quality of developed complementary diet and evaluate its potentials to manage protein energy malnutrition. This was approached through the exploitation of available local resources such as sorghum, soybeans, and orange‐fleshed sweet potatoes which are cheap, locally available and are of high nutritive value.

## 2 MATERIALS AND METHODS

Sorghum *(Sorghum bicolour)* and soybean *(Glycine max)* were obtained from *Oja Oba* market in Oka Akoko, Akoko South West local government area of Ondo State, Nigeria, while the orange‐fleshed sweet potato tubers (*Ipomoea batatas L*.) were obtained from Osun State Ministry of Agriculture, Osogbo, Osun State, Nigeria. The food materials were authenticated at the Department of Crop, Soil and Pest Management, Federal University of Technology Akure, Nigeria. Cerelac (a commercial complementary formula) used as a reference sample was purchased from Oba market Akure, Nigeria. Albino Wister rats were obtained from animal house, Department of Biochemistry, Federal University of Technology, Akure, Nigeria. All chemicals and reagents used in the study were of analytical grade and were obtained from Sigma‐Aldrich, London, United Kingdom.

### 2.1 Samples preparation

#### 2.1.1 Production of sorghum flour

The sorghum flour was produced by the method described by Adebayo‐Oyetoro et al. (2012). The grains were sorted and manually cleaned to remove broken seeds, dust, and other extraneous materials. A portion was spontaneously fermented by submerged fermentation in clean water for 72 hr, after which it was drained and oven‐dried (Plus11 Sanyo Gallenkamp PLC, UK) at 65°C for 24 hr. Dried fermented and unfermented sorghum were milled separately into flour using laboratory hammer mill (Fritsch, D‐55743; Idar‐oberstein‐Germany), sieved (250‐μm screen), packaged, sealed, and stored in plastic container at room temperature (25°C ± 2) until use.

#### 2.1.2 Production of soybean flour

The soybean flour was prepared using the method described by Osundahunsi (2006) with slight modification. Soybean grains were sorted, while extraneous materials were removed. A portion was submerged in clean water and fermented for 72 hr after which it was washed, drained, and oven‐dried (Plus11 Sanyo Gallenkamp PLC, UK) at 65°C for 24 hr. The fermented and unfermented dried samples were roasted separately at 115°C for 10 min, dehulled, winnowed, milled into flour using laboratory hammer mill (Fritsch, D‐55743, Idar‐oberstein‐Germany), sieved (using the 250 μm screen), packaged, sealed, and stored in plastic container at room temperature (25^o^C ± 2) until use.

#### 2.1.3 Production of orange‐fleshed sweet potato flour

Orange‐fleshed sweet potato (OFSP) flour was prepared following the method described by Nnam (2001) and Oloo et al. (2014) with slight modification. The tubers were sorted, washed thoroughly with clean water, peeled, and sliced into 2 mm thickness using electric slicer. The OFSP slices were immersed into 0.25% sodium metabisulphite for 5 min to prevent browning reactions (do Nascimento & Canteri, 2019). A portion of OFSP was fermented by submerging in clean water for 72 hr after which it was drained. The fermented and unfermented OFSP were oven‐dried (Plus11 Sanyo Gallenkamp PLC, UK) separately at 65°C for 24 hr and then milled into flour using laboratory hammer mill (Fritsch, D‐55743; Idar‐oberstein‐Germany), sieved, packaged, sealed, and stored in plastic container at room temperature (25°C ± 2) until use.

### 2.2 Formulation of blends

The composite blends were developed using the mixture design of the Response Surface Methodology design software (Design expert version 10.0.). The upper limits used in the study were sorghum (80%), soybean (40%), and orange‐fleshed sweet potato (40%), while the lower limits were 50%, 10%, and 10%, respectively. Sixteen (16) runs were generated and used to obtain optimal blends using the proximate, mineral, and antinutrient composition as responses. Besides, moisture content that was minimized, all other responses were maximized while setting the optimization goals. The optimal blends were selected based on high protein, zinc, iron, and vitamin A contents and was then used in this study (Table 1).

**Table 1:** The response surface methodology variables conditions and responses

Runs	Sorghum flour (%)	Soy flour (%)	OFSP flour (%)	Moisture (%)	Ash (%)	Fat (%)	Fiber (%)	Protein (%)	CHO (%)	Fe (mg/100 g)	Zn (mg/100 g)	Vit. A (mg/100 g)
1	65	10	25	4.47	4.05	12.26	1.87	13.50	63.86	4.95	3.04	12.73
2	70	20	10	5.45	4.75	14.08	1.29	17.52	56.91	6.83	4.21	13.33
3	80	10	10	4.74	3.33	12.48	1.28	13.51	64.69	3.86	4.74	11.80
4*	**56**	**17**	**27**	**5.09**	**6.41**	**14.11**	**1.73**	**10.53**	**62.13**	**7.23**	**2.69**	**14.22**
5	50	25	25	6.94	4.10	15.70	1.83	18.74	52.68	6.51	2.96	12.30
6	65	25	10	5.29	2.74	15.41	1.40	18.75	55.72	5.24	3.44	13.88
7	50	10	40	5.99	2.83	14.43	2.30	13.5	60.96	6.18	3.81	12.38
8	55	15	30	4.20	5.88	14.93	2.19	15.25	57.56	5.22	6.87	13.34
9	50	10	40	5.93	2.89	14.43	2.34	13.51	60.90	6.14	3.74	2.50
10	50	40	10	5.10	4.72	21.24	1.44	26.24	41.27	8.37	2.11	12.70
11	80	10	10	4.75	3.36	12.50	1.26	13.50	63.12	3.88	4.76	12.19
12	50	30	20	5.64	3.98	17.31	1.74	20.53	50.81	7.86	3.23	12.05
13	50	10	40	5.95	2.67	14.45	2.38	13.52	61.03	6.16	3.8	12.24
14	65	10	25	4.51	4.10	12.22	1.86	13.53	63.79	4.96	3.02	12.70
15	50	40	10	5.08	4.73	21.13	1.44	26.24	41.38	8.36	2.15	12.50
16	59	31	10	5.65	4.18	17.07	1.42	20.38	51.3	7.21	4.13	11.51

Note

Keys: where CHO = Carbohydrates; OFSP = Orange‐fleshed sweet potato; Fe = Iron; Zn = Zinc, Vit. A = Vitamin A.

### 2.3 Determination of proximate composition

The proximate composition (moisture, crude fat, crude fiber, crude ash, and crude protein) of flour blends, ogi, and cornstarch was all determined according to AOAC (2012), while the carbohydrate content was calculated by difference from 100. The energy value was calculated using the Atwater factor as described by Zou et al. (2007).

### 2.4 Animal experimental study

Forty‐nine healthy weanling albino Wistar rats weighing between 20 and 25 g were obtained from the Department of Biochemistry Animal Laboratory, Federal University of Technology Akure. The study was approved by the Federal University of Technology Akure ethical committee under the ethical number FUTA/SAAT/2016/015. The rats were randomly distributed in metabolic cages and fed with normal rat pellets for a period of 7 days for proper acclimatization before commencement of the experiments. Animals receive lighting regimen of 12‐hr light and 12‐hr darkness at room temperatures (25°C ± 2) and were administered feed and water ad libitum. Diet was formulated based on 10% iso‐nitrogenous protein levels. After the acclimatization period, the animals were reweighed and grouped into seven groups of seven rats per group. Rat groups were assigned to various diets as shown below.

#### 2.4.1 Experimental groups

*Ogi*: Weanling rats fed *ogi* only (internal negative control)
CT: Weanling rats fed cerelac – commercial sample only (positive control)
Cornstarch: Weanling rats fed basal diet only (negative control)
UF2: Weanling rats fed unfermented sorghum (56%)‐soybean (17%)‐OFSP (27%) diet only
UF3: Weanling rats fed unfermented sorghum (59%)‐soybean (31%)‐OFSP (10%) diet only
F3: Weanling rats fed fermented sorghum (56%)‐soybean (17%)‐OFSP (27%) diet only
F3: Weanling rats fed fermented sorghum (59%)‐soybean (31%)‐OFSP (10%) diet only

The rats were weighed at 4‐day intervals for 28 days of the experiment, and weight gained or loss was calculated. Urine was collected from each cage in a small urine container, which contain about 1 ml concentrated sulfuric acid. Similarly, fecal samples were collected on the last 7 days. The fecal samples were weighed, dried, and milled prior to laboratory analysis (Osundahunsi & Aworh, 2003). The feces and urine samples were separately analyzed in triplicate for percent nitrogen levels. The animals were anathesized and the blood samples collected through cardiac heart puncture using appropriate syringe and needles. The blood samples of the animals were collected in non‐heparinized collection tube and heparinized tubes for serum chemistry and blood parameters analysis, respectively. The following parameters: packed cell volume (PCV), hemoglobin concentration (HBC), red blood cell count (RBC) and white blood cells (WBC), and thrombolytic indicators, were evaluated as described by Princewill‐Ogbonna et al. (2015). Hemoglobin concentration (HBC) was estimated using the cyanomethemoglobin method, and mean corpuscular hemoglobin concentration (MCHC), mean corpuscular hemoglobin (MCH), and mean corpuscular volume (MCV) were calculated.

The serum was obtained by centrifugation at 3,000 × *g* and was analyzed for serum chemistry parameters such as alanine aminotransferase (ALT), aspartate aminotransferase (AST), and alkaline phosphatase (ALP) levels were determined by the method of Reitman‐Frankel as described by Reitman and Frankel (1957) while urea (Fawcett & Scott, 1960), albumin (Weiss et al., 1965) and total protein levels (Gornall et al., 1949). Globulin levels were calculated by difference, while creatinine levels were determined using test kit (Randox Laboratories, Antrim, UK). Animals were sacrificed by cervical dislocation, and organs were excised. Weight of organs was determined across group using standard techniques. The nitrogen intake and excreted in feces and urine of animals were determined and were used to calculate the nutritional quality indices; net protein retention (NPR), biological value (BV), nitrogen retention (NR), feed efficiency (FE), net protein utilization (NPU), true digestibility (TD), and protein efficiency ratio (PER) were calculated as described in the equations below (Chapman et al., 1959; Friedman, 1996; Miller & Payne, 1961).

(1) $$\text{NR}=N_{i}‐\left(N_{f}‐N_{\mathrm{ef}}\right)‐\left(N_{u}‐N_{\mathrm{eu}}\right)$$

(2) $$\text{BV}=\frac{N_{i}‐\left(N_{f}‐N_{\mathrm{ef}}\right)‐\left(N_{u}‐N_{\mathrm{eu}}\right)}{N_{i}‐\left(N_{f}‐N_{\mathrm{ef}}\right)}\times 100$$

(3) $$\text{FE}=\frac{\text{Weight gained}}{\text{Food intake}}$$

(4) $$\text{NPU}=\frac{N_{i}‐\left(N_{f}‐N_{\mathrm{ef}}\right)‐\left(N_{u}‐N_{\mathrm{eu}}\right)}{N_{i}}\times 100$$

(5) $$\text{PER}=\frac{\text{Weight gained}}{\text{Protein intake}}$$

(6) $$\text{TD}=\text{PER}\times \text{Daily intake of protein}\left(g\right)$$

where *N_i_* = nitrogen intake of the test diet; *N_f_* = fecal nitrogen of the test diet; *N_ef_* = fecal nitrogen excreted; *N_u_* = urinary nitrogen of the test diet; *N_eu_* = urinary excreted nitrogen (endogenous nitrogen).

### 2.5 Statistical analysis

Determinations were in triplicate, and data were analyzed using analysis of variance (ANOVA) in the Statistical package for Social Sciences software version 21. The test for significance was determined by separating the means using Duncan new multiple range test (DNMRT) at *p* ≤ .05.

## 3 RESULTS AND DISCUSSION

### 3.1 Proximate composition of formulated complementary food blends

As presented in Table 1, sixteen runs were generated and the runs, moisture contents ranged from 4.20% to 6.94% and were highest in run 5 and lowest in run 8, while the ash content ranged from 2.67% to 6.41% and was highest in run 4. The crude fat content ranged from 12.22% to 21.24%, while the crude fiber content ranged from 1.26% to 2.38%. Runs 10 and 15 had the highest and similar amount of crude protein (26.24%) compared to other runs. The essential micronutrients such as iron, zinc, and vitamin A are important components in complementary foods since they help in the proper development and functioning of the brain and the body of infants. The iron content ranged from 3.86 to 8.37 mg/100 g, zinc (2.11–6.87 mg/100 g) and vitamin A (2.50–14.22 mg/100 g). Based on the highest desirability, high protein, iron, zinc, and vitamin A and values within the Recommended Dietary Allowance (RDA), run 4 (Sorghum flour 56%; soybean flour 17%; OFSP 27%) was selected as an optimal blend and was further used in the study. The proximate composition of fermented and unfermented sorghum, soybean, and orange‐fleshed sweet potato flour blends is presented in Table 2. Proximate composition is an important criterion to determine the nutrient content and quality of foods (Qayyum et al., 2012). The moisture content of developed blends ranged from 2.5% to 9.24%. The formulated blends were significantly different from one another at (*p* ≤ .05) with UF3 having the lowest, while F2 had the highest value. Fermentation process increased the moisture content of the developed complementary foods as shown by increased moisture in fermented samples compared to the unfermented. The high moisture content obtained in *ogi* (8.31%) was expected and supports the previous value (7.82%) reported by Osundahunsi and Aworh (2002) in a study involving the development of weaning diet from tempe. The moisture content of samples increased with increasing sorghum incorporation in the samples. The moisture content of samples was above the recommended values for moisture (<5%) in complementary foods (FAO/WHO, 1991). The high moisture content obtained in this study shows that the product may not have a long shelf life. High moisture in food is indicative of increased predisposition to microbial attack and invariably shortening the shelf life of food product (Adebayo‐Oyetoro et al., 2012). However, according to the report of Kumolu‐Joh and Ndimele (2011) moisture content in excess of 14% in flours has greater susceptibility to bacteria and mold growth.

**Table 2:** Proximate composition and energy value of formulated complementary foods (Dry weight basis)

Sample	Moisture (%)	Ash (%)	Fat (%)	Fiber (%)	Protein (%)	CHO (%)	Energy (Kcal)
UF2	8.42 ± 0.08^b^	2.75 ± 0.05^d^	13.94 ± 0.06^c^	3.17 ± 0.06^a^	13.21 ± 0.14^f^	58.51 ± 0.41^d^	417.14 ± 1.04^b^
UF3	7.23 ± 0.11^d^	3.71 ± 0.06^a^	15.24 ± 0.14^a^	2.85 ± 0.03^b^	18.66 ± 0.14^b^	52.31 ± 0.15^f^	426.28 ± 1.14^a^
F2	9.24 ± 0.06^a^	2.53 ± 0.06^f^	12.34 ± 0.06^e^	2.62 ± 0.09^c^	15.80 ± 0.03^d^	57.47 ± 0.07^e^	409.22 ± 0.14^e^
F3	7.43 ± 0.08^c^	3.17 ± 0.02^c^	14.64 ± 0.06^b^	2.43 ± 0.06^d^	25.29 ± 0.07^a^	47.04 ± 0.11^g^	428.16 ± 1.15^a^
OGI	8.31 ± 0.57^b^	1.09 ± 0.01^h^	5.17 ± 0.11^h^	0.85 ± 0.01^f^	7.09 ± 0.31^g^	77.49 ± 0.21^a^	420.41 ± 0.54^d^
CT	2.50 ± 0.10^g^	3.23 ± 0.21^b^	10.28 ± 0.25^g^	2.22 ± 0.11^e^	15.79 ± 0.17^c^	65.98 ± 0.17^b^	422.12 ± 0.34^c^
*RDA	<5	<3	10–25	<5	>15	64	400–425

Note

Values are means ± standard deviation, samples within the same column with different superscripts are significantly different (*p* ≤ .05). Key: OFSP = Orange‐fleshed Sweet Potato; UF2 = Unfermented sorghum (56%)–soybean (17%)–OFSP (27%); F2 = Fermented sorghum (56%)–soybean (17%)–OFSP (27%); UF3 = Unfermented sorghum (59%)–soybean (31%)–OFSP (10%); F3 = Fermented sorghum (59%)–soybean (31%)–OFSP (10%); CT = Cerelac, *RDA—Recommended values for infant complementary foods (FAO/WHO, 1991).

The ash content is a measure of the total amount of mineral present in food. The ash content of samples ranged from 1.09% to 3.71% and was significantly different from one another (*p* < .05). The ash content was highest in UF3 (3.71%) and lowest in *ogi* (1.09%). Fermentation process negatively affects the ash content of samples. The ash content of samples was within the recommended value (<3%) for complementary foods (FAO/WHO, 1991). The high ash content obtained in UF3 could be due to the larger soybean incorporation in the sample and no fermentation treatment as compared to F3. Ash content has been described to be a function of legume supplementation (Nnam, 2001) similar to observation in this study. High ash content in samples implies a rich sources of inorganic nutrients (Ndife et al., 2013). The decrease in the ash content after fermentation as obtained in this study could be due to leaching of the soluble inorganic salt during fermentation (Aluge et al., 2016) and was contrary to that reported in fermented sweet potato as reported by Nnam (2001). This variation could be due to variation in the type of sweet potato used in this study.

The fat content of samples ranged from 10.28% to 15.24% and was significantly different at *p* ≤ .05. The crude fat content was highest in UF3, while control sample (CT) had the lowest fat content. The crude fat content was within the RDA values for complementary foods (FAO/WHO, 1991). The fat content of sample increased with increasing supplementation with soybean. This observation is expected as soybean is known to be high in fat (Choi & Rhee, 2006). Fermented samples had lower fat content compared to the unfermented. The decrease in fat content observed in the fermented samples could be attributed to the activities of colonizing microorganism and lipolytic enzymes during fermentation (Ijarotimi & Keshinro, 2013). Low fat content in samples could result in increased shelf life due to reduced chances of rancidity (Akinola et al., 2017).

Fiber is nondigestible carbohydrate originating from plants, aids the improvement of the heart condition, reduces risk of chronic diseases, and promotes gastrointestinal tract digestion and bowel movement (Anderson et al., 2009; Moshfegh et al., 2005). High fiber content in foods is associated with increased moisture retention (Haruna et al., 2011), and it is undesirable in infant because of the physiological make‐up of their gastrointestinal tract (Oyarekua & Adeyeye, 2009). The fiber content of all the samples ranged from 0.85% to 3.17%. The highest fiber content was obtained in UF2 and lowest in *ogi*. The lower fiber content obtained in the fermented samples could be due to the depletion of sugars and dietary fibers during fermentation process. Similarly, the incorporation of OFSP decreased crude fiber content of the samples. Hence, fermentation and increasing substitution of fermented and unfermented sorghum flour with OFSP result in a decreased fiber content. However, the obtained values were within the recommended value (<5%) for complementary foods as stipulated by FAO/WHO (1991). Low fiber content of complementary foods reduces bulkiness of food and thus encourages high digestibility and absorption of essential nutrients such as proteins and minerals in children (Adegbanke et al., 2018; Cuddeford et al., 1995). There was no significant difference in the fiber content of cerelac and F2. Low fiber content in complementary food is desirable as its enables children to consume more food giving a greater opportunity to meet the daily energy and other vital nutrient requirements. The low crude fiber obtained supports the previous report in complementary food developed from sorghum–sesame–carrot–crayfish (Onabanjo et al., 2009) and fermented sorghum–cowpea (Oyarekua & Adeyeye, 2009).

The protein contents of the blends ranged from 7.09% to 25.29% and were highest in F3 (25.29%) and lowest in ogi (7.09%). The low protein content obtained in ogi was expected as cereals are known as a poor source of protein (Belton & Taylor, 2004). The protein content of the fermented blends was higher than that in unfermented blends which could be due to the metabolic activities of microorganisms resulting in the release of extracellular enzymes (Akinola & Osundahunsi, 2017; Oboh & Akindahunsi, 2004). The increase in protein content as obtained in this study agrees with the report of Afoakwa et al. (2004) on the increase in protein contents and product functionality due to fermentation. Soybean substitution in the samples improved the crude protein content as shown in UF3 (18.66%) and F3 (25.29%) compared to other samples. The protein content obtained in samples was above the RDA values (˃15%) for complementary foods (FAO/WHO, 1991) except UF2 and *ogi*. This study supports the previous report of Osundahunsi and Aworh (2002) on low protein content of *ogi* than the test samples. Nutritionally, high protein content in foods is beneficial for growth and development of infants. Essential nutrient like proteins is needed for biochemical activities, building, repair of body tissues, and organs (Oyarekua, 2010). Fermentation increases the digestibility of plant proteins (Akinola et al., 2017; Hag et al., 2002; Pranoto et al., 2013). Fermentation process has advantage over other processing methods, which includes its ability to destroy protease (trypsin) inhibitors (Osman, 2011) and cause a partial predigestion of grain proteins during fermentation (Day & Morawicki, 2016).

Carbohydrate is the main source of energy in the human body. The carbohydrate content of samples ranged from 47.04% to 77.49% and was significantly different at *p* ≤ .05. The *Ogi* samples had the highest carbohydrate content compared to others. This observation may be due to the fact that ogi is mainly composed of starch. The substitution of fermented and unfermented sorghum influenced the reduced carbohydrate content observed in this study. The unfermented samples had the highest carbohydrate content compared to the fermented. The reduced carbohydrate content in fermented samples could be due to the utilization of fermentable sugars by lactic acid bacteria either for growth and other metabolic activities (Akinola & Osundahunsi, 2017). Fermentation activates starch‐hydrolyzing enzymes such as α‐amylase and maltase which degrade starch into maltodextrins and simple sugars (Osman, 2011). The glucose released during fermentation is a preferred substrate for fermentative microorganisms in foods and could partly explain the decrease in total carbohydrate content after fermentation (Osman, 2011). Energy values of the blends ranged from 409.22 to 428.16 kCal and were within the recommended range of values for infant complementary foods (FAO/WHO, 1991). Sample F3 had the highest energy value, and this indicates that infant may consume less quantity of the complementary food to meet the daily energy requirement.

### 3.2 Growth parameters of experimental rat fed formulated diets

**Figure 1:**
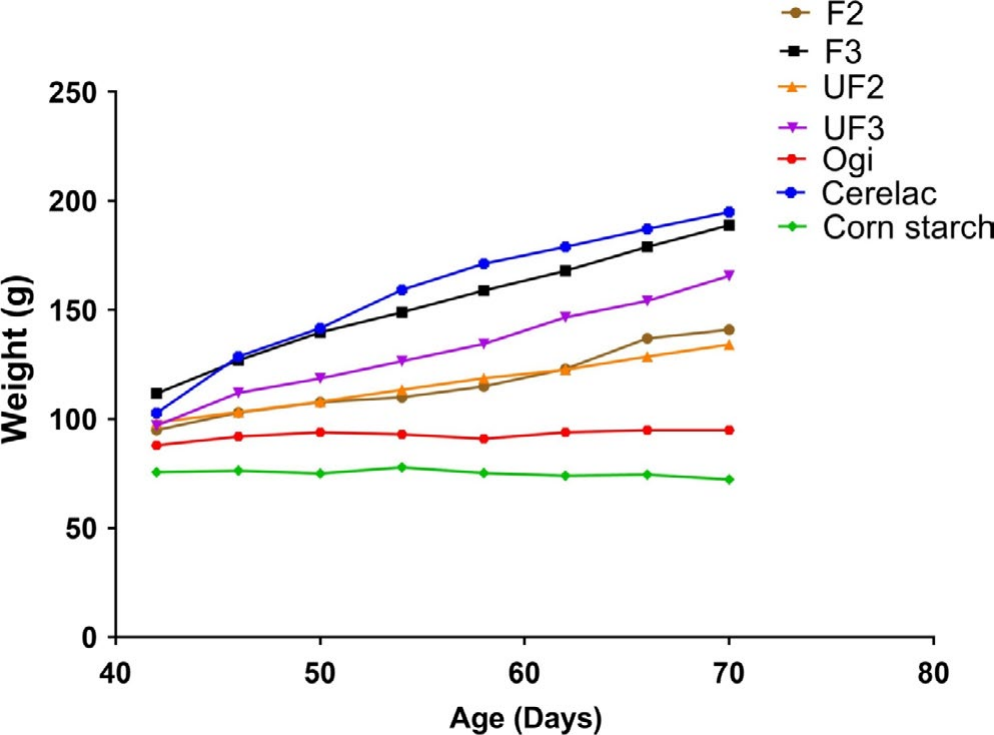
Weight‐for‐age (underweight) of rats fed unfermented and fermented complementary diet

**Figure 2:**
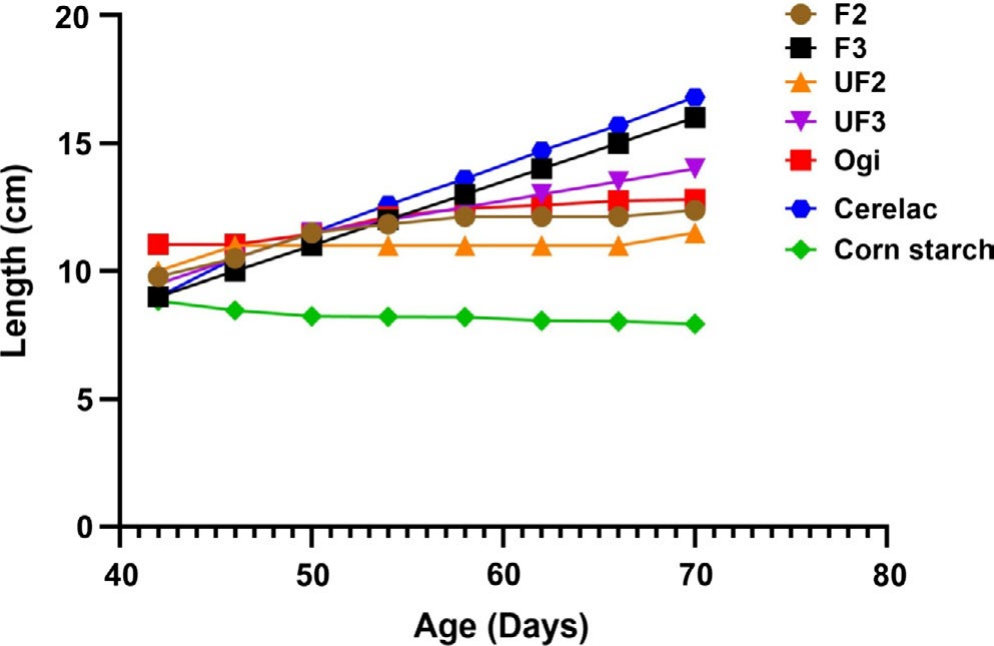
Length‐for‐age (stunting) of rats fed unfermented and fermented complementary diet

The growth pattern of experimental rat placed on formulated blends and control samples is presented in Figures 1 and 2. The nutritional status of the experimental rats showed that the rats fed with F3 had better growth performance and were comparable to those rats fed cerelac (CT), but significantly higher compared with other formulated blends (F2, UF2, UF3), ogi, and corn starch. The rats fed F3 had the highest growth performance among rats fed the developed food samples with reference to weight‐for‐age and length‐for‐age. Nutritionally, from this present study, it could be deduced that the formulated food sample (F3) may be suitable as a complementary food, especially to support infant growth and development and prevent malnutrition among under‐five aged children. However, acceptability of this product through sensory evaluation is important prior to commercialization.

### 3.3 Nutritional quality and relative organ weight of experimental rats fed formulated diet

The protein quality and relative organ weight of experimental rats fed on formulated diets and control samples are showed in Table 3. The weight gained by rat fed formulated diets ranged from 46.00 to 77.25 g and was highest in F3 group and lowest in UF2, and the weight gained was higher than groups fed ogi (7.00 g) and corn starch (3.34 g), but lower to the commercial sample (cerelac). The food intake of animals ranged from 355.09 to 684.76 g and increased with fermentation of the samples and was higher than that obtained in group fed *ogi* and corn starch. This observation might be due to improve palatability of the diet due to fermentation. Therefore, the developed diet improves food intake by the rats, although not as high as in cerelac (684.76 g). The feed efficiency ratio (FER) ranged from 0.01 to 0.14 g and was significantly different across groups (*p* ≤ .05). The FER of developed diet was higher than *ogi* and corn starch. The nitrogen retention (NR) and true digestibility (TD) ranged from 3.86 to 18.91 g and 35.43 to 95.25%, respectively. The NR increased with fermentation and soybean supplementation in the diet and was highest in F3 and lowest as expected in the basal diets (ogi and corn starch). The NR of group F3 was higher than the commercial sample (cerelac) thus signifying the potential of F3 as a substitute for cerelac in weaning of infants. The net protein utilization (NPU) ranged from 17.58% to 95.25%, while the NPU for formulated diet was higher than *ogi* (59.20%) and corn starch (17.58%) but lower than cerelac (95.25%). The corrected value of protein efficiency ratio (PER) based on 2.5 for cerelac showed that UF2 sample (2.25) has the least, while F3 (2.48) has the highest, and PER of the formulated diets was lower than FAO and WHO (1989), recommended values of 2.7 for an ideal food.

**Table 3:** Nutritional qualities of formulated complementary diets

Parameters	UF2	UF3	F2	F3	*Ogi*	Corn starch	Cerelac
Weight gained (g)	35.75^e^	68.75^c^	46.00^d^	77.25^b^	7.00^f^	3.34^g^	92.25^a^
Food intake (g)	577.39^d^	552.76^e^	581.45^c^	596.74^b^	453.23^f^	355.09^g^	684.76^a^
Feed efficiency ratio	0.06^e^	0.12^c^	0.08^d^	0.13^b^	0.02^f^	0.01^g^	0.14^a^
Nitrogen retention	9.37^e^	10.26^d^	14.51^b^	18.91^a^	3.86^f^	0.00^g^	14.44^c^
True digestibility (%)	72.05^e^	78.29^d^	80.87^c^	84.44^b^	61.65^f^	35.43^g^	95.25^a^
Net protein utilization	71.35^e^	77.06^d^	83.63^c^	80.40^b^	59.20^f^	17.58^g^	95.25^a^
Biological value	75.11^e^	75.87^d^	78.40^c^	78.44^b^	51.89^f^	33.84^g^	93.48^a^
Protein efficiency ratio	2.74^c^	2.90^b^	2.86^b^	3.03^a^	2.51^d^	1.76^e^	3.05^a^
*Relative organ weight (g)*							
Liver	0.85^e^	0.90^d^	1.12^c^	1.37^a^	1.13^c^	0.65^f^	1.35^b^
Kidney	0.21^d^	0.21^d^	0.24^c^	0.28^a^	0.20^e^	0.20^e^	0.25^b^
Heart	0.53^e^	0.56^d^	0.60^c^	0.65^b^	0.49^f^	0.32^g^	0.69^a^

Note

Values are means ± standard deviation, samples within the same column with different superscripts are significantly different (*p* ≤ .05).

Key: OFSP = Orange‐fleshed Sweet Potato; UF2 = Unfermented sorghum (56%)–soybean (17%)–OFSP (27%); F2 = Fermented sorghum (56%)–soybean (17%)–OFSP (27%); UF3 = Unfermented sorghum (59%)–soybean (31%)–OFSP (10%); F3 = Fermented sorghum (59%)–soybean (31%)–OFSP (10%); CT = Cerelac; Corn starch = Basal diet.

The biological values (BV) of formulated diets ranged from 75.11% to 78.44% and were highest in F3 and lowest in UF2. Cerelac fed group had the highest BV, while the corn starch fed group had the lowest (33.84%) BV. The BV of the formulated diets were above FAO/WHO, (1989) recommended values of 70% for an ideal food thus indicating that the protein content of the formulated diets may adequately provide the necessary protein requirements to support growth and development in both infants and adults. Biological value (BV) is a measure of the proportion of absorbed protein from a food, which becomes incorporated into the proteins of the organism's body (Černá, 2011). BV also describes how readily the digested protein can be used in cell protein synthesis of an organism.

The relative organ (liver, kidney, and heart) weight of the experimental animals fed the formulated diets ranged from 0.85 to 1.37 g, 0.21 to 0.28 g, and 0.53 to 0.65 g, respectively. These values were higher than organ weight: Liver (1.13 g; 0.65 g), kidney (0.20 g; 0.02 g), and heart (0.49 g; 0.32 g) recorded in *ogi* and corn starch fed group, respectively. These observations further showed that the formulated diets were better in protein quality and may be suitable as a complementary food to support growth of children. This finding agreed with the report on the nutritional qualities of foods that were formulated from the combinations of two or more plant‐based food materials (Ijarotimi & Keshinro, 2012; Okpala & Okoli, 2011). Similarly, Osundahunsi and Aworh (2003) reported a lower weight for some vital organs in rats fed with the basal diet singly which is an indication of some forms of abnormal development thereby corroborating the low organ weight obtained in *ogi* and corn starch fed groups in this study.

### 3.4 Hematological properties of experimental rats placed on formulated diet

Hematological properties of the experimental rats fed the formulated and control diets are presented in Table 4. The packed cell volume (PCV), hemoglobin concentration (HbC), and white blood cells (WbC) ranged from 9.00% to 22.50%, 4.00 to 7.90 g/dl, and 4.90 to 8.70 x 10^3^ mm^3^, respectively. The red blood concentration (RbC) and the lymphocytes count ranged from 0.64 to 2.45 × 10^3^ mm^3^ and 23.00%–68.60%, respectively. The PCV, Hbc, RBC, and lymphocytes values of F3 group were highest among the rats groups fed formulated diet and were lower compared to commercial weaning food (cerelac). The high concentration of PCV, HbC, RBC, and lymphocytes of the experimental rats fed on sample F3 and cerelac (positive control) further established nutritional quality of these products. This finding agrees with the report of Roberts et al. (2000) who established that diets containing quality protein and iron usually enhance production of hemoglobin and immunity in animals. In contrary, low PCV and Hbc that were observed in the other formulated diets (UF2, UF3, and F2) may lead to poor production of hemoglobin and, hence, could cause anemia (Ijarotimi & Keshinro, 2012; Osundahunsi & Aworh, 2002). The values of the mean cell hemoglobin concentration (MCHC), mean cell hemoglobin (MCH) mean cell volume (MCV) and neutrophils of the experimental rats fed on the formulated diets, corn starch (basal diet), ogi (negative control), and cerelac (positive control) ranged from 22.15–34.20 g/dl, 18.67–38.40 pg, and 15.43 to 98.50 fl and 13.90% to 40.40% respectively. The MCHC, MCH, and MCV are useful indices of the average Hb concentration of red blood cells, and low concentration of this hematological parameter in animals is indicative of hemolytic anemia, while an increase in these parameters is suggestive of massive intravascular hemolysis (Sridhar Prasad et al., 2018).

**Table 4:** Hematological parameters of rats fed formulated complementary diets

Parameters	UF2	UF3	F2	F3	Cornstarch	*Ogi*	Cerelac
PCV (%)	21.00 ± 0.09^b^	20.00 ± 0.12^c^	21.00 ± 0.06^b^	22.50 ± 0.30^a^	9.00 ± 0.09^e^	22.00 ± 0.49^a^	22.00 ± 0.36^a^
Hb (g/dl)	7.00 ± 0.03^b^	6.70 ± 0.04^c^	7.00 ± 0.04^ab^	7.50 ± 0.05^a^	4.00 ± 0.10^e^	7.30 ± 0.60^a^	7.90 ± 0.75^a^
WBC (×10^3^ mm^−3^)	4.90 ± 0.04^f^	6.40 ± 0.03^c^	7.10 ± 0.02^b^	8.70 ± 0.14^a^	5.60 ± 0.22^d^	5.20 ± 0.03^e^	8.20 ± 0.43^a^
RBC (×10^3^ mm^−3^)	2.35 ± 0.02^c^	2.20 ± 0.01^d^	2.00 ± 0.01^e^	3.00 ± 0.07^a^	0.64 ± 0.00^g^	2.45 ± 0.05^b^	2.45 ± 0.01^b^
MCHC (g/dl)	33.30 ± 0.31^b^	33.50 ± 0.31^ab^	33.30 ± 0.37^b^	34.20 ± 0.40^a^	22.15 ± 0.12^c^	31.10 ± 0.06^c^	33.10 ± 0.37^b^
MCH (pg)	29.80 ± 0.26^d^	30.40 ± 0.24^c^	30.00 ± 0.64^c^	38.40 ± 0.59^a^	18.67 ± 0.20^g^	23.90 ± 0.12^e^	34.30 ± 0.21^b^
MCV (fl)	89.30 ± 0.61^c^	90.1 ± 0.42^b^	90.00 ± 0.47^b^	98.50 ± 0.38^a^	15.43 ± 0.17	81.30 ± 0.95^d^	89.90 ± 0.59^c^
Neutrophils (%)	29.00 ± 0.32^e^	30.00 ± 0.20^d^	35.00 ± 0.51^b^	40.40 ± 0.54^a^	13.90 ± 0.05^g^	30.70 ± 0.12^d^	32.00 ± 0.71^c^
Lymphocytes	59.00 ± 0.47^d^	62.00 ± 0.51^c^	63.00 ± 0.42^b^	68.60 ± 0.19^a^	23.00 ± 0.26^g^	55.00 ± 0.43^e^	67.00 ± 1.32^a^
Monocytes (%)	1.00 ± 0.00^c^	0.00 ± 0.00^d^	2.00 ± 0.00^b^	3.20 ± 0.00^a^	0.00 ± 0.00^d^	2.00 ± 0.00^b^	3.00 ± 0.20^ab^
Eosinophils (%)	0.00 ± 0.00^b^	0.00 ± 0.00^b^	0.00 ± 0.00^b^	1.00 ± 0.00^a^	0.00 ± 0.00^b^	0.00 ± 0.00^b^	0.00 ± 0.00^b^
Basophils (%)	0.00 ± 0.00^a^	0.00 ± 0.00^a^	0.00 ± 0.00^a^	0.00 ± 0.00^a^	0.00 ± 0.00^a^	0.00 ± 0.00^a^	0.00 ± 0.00^a^

Note

Values are means ± standard deviation, samples within the same column with different superscripts are significantly different (*p* ≤ .05). Keys: OFSP = Orange‐fleshed Sweet Potato; UF2 = Unfermented sorghum (56%)–soybean (17%)–OFSP (27%); F2 = Fermented sorghum (56%)–soybean (17%)–OFSP (27%); UF3 = Unfermented sorghum (59%)–soybean (31%)–OFSP (10%); F3 = Fermented sorghum (59%)–soybean (31%)–OFSP (10%); CT = Cerelac; Corn starch = Basal diet, PCV = Packed cell volume, Hb = Hemoglobin, WBC = White blood cell, RBC = Red blood cell, MCHC = Mean corpuscular hemoglobin concentration, MCV = mean cell volume, MCH = Mean corpuscular hemoglobin.

### 3.5 Serum biochemical parameters of experimental rats fed with formulated diet

Serum biochemical parameters of experimental rat treated with formulated diet, basal diet, and control samples are shown in Table 5. The total blood protein ranged from 1.27 to 6.04 g/dl and albumin concentration (1.04–4.40 g/dl). The total blood protein was highest in rats fed cerelac and was comparable to group F3. The serum albumin concentration was highest in rats fed F3 diet (4.12 g/dl) and was comparable to the commercial weaning food (cerelac). The serum protein and albumin concentrations reported in this study were higher than the normal ranges (6–8 g/dl; 3.5–5.0 g/dl, respectively) reported by Giannini et al. (1999) and Diana Nicoll (2007). The urea nitrogen and the creatinine of rats fed with the formulated diets, corn starch, *ogi,* and cerelac ranged from 3.58 to 15.32 mg/dl. The values were all within the normal ranged reported by Iseki et al., (1997) except the experimental rats fed on starch. Fermentation had a significant reduction in the blood urea of animals exposed to the formulated diets. Healthy kidney remove creatinine and urea nitrogen from the blood, the higher the creatinine and urea value the less effective the kidney function (Rusul Arif & Haider, 2014). Creatinine and urea concentration were lower compared to other formulated diets. Thus, the lower urea and creatinine concentration obtained in the fermented diets implies that the formulated diets F3 had no negative effect on the kidney function.

**Table 5:** Biochemical properties of rats fed on control samples and formulated diet

Samples	Albumin (g/dl)	Total protein (g/dl)	Globulin (g/dl)	Urea (mg/dl)	Creatinine (mg/dl)	AST (U/L)	ALP (U/L)	ALT (U/L)	AST/ALT
UF2	3.07 ± 0.12^c^	3.40 ± 0.82^c^	0.33 ± 0.01^c^	14.75 ± 5.71^b^	6.15 ± 0.21^a^	20.86 ± 0.57^b^	41.41 ± 0.32^d^	40.71 ± 0.07^b^	0.51
UF3	3.52 ± 0.17^b^	3.57 ± 0.51^b^	0.05 ± 0.04^e^	11.56 ± 2.04^c^	5.63 ± 0.79^b^	20.43 ± 0.41^b^	51.06 ± 0.43^c^	50.44 ± 0.13^a^	0.41
F2	3.72 ± 0.77^ab^	5.62 ± 0.82^ab^	1.90 ± 0.21^a^	10.16 ± 1.40^d^	4.80 ± 0.95^bc^	19.30 ± 0.43^c^	61.33 ± 0.32^b^	50.46 ± 0.21^a^	0.38
F3	4.40 ± 0.93^a^	5.94 ± 0.89^a^	1.54 ± 0.02^b^	9.16 ± 0.98^e^	4.76 ± 0.34^c^	19.39 ± 0.27^c^	64.30 ± 0.65^a^	50.75 ± 0.21^a^	0.38
Cornstarch	1.04 ± 0.16^e^	1.27 ± 0.55^e^	0.23 ± 0.03^d^	3.58 ± 0.82^g^	1.56 ± 1.51^e^	15.17 ± 0.31^d^	23.12 ± 0.51^e^	30.43 ± 0.44^c^	0.49
*Ogi*	3.02 ± 0.78^bc^	3.04 ± 0.19^d^	0.02 ± 0.01^e^	15.32 ± 0.23^a^	6.14 ± 0.23^a^	20.43 ± 0.47^b^	41.12 ± 0.95^d^	40.69 ± 0.65^b^	0.50
Cerelac	4.12 ± 0.81^a^	6.04 ± 0.89^a^	1.92 ± 0.05^a^	7.77 ± 0.77^f^	2.07 ± 0.10^d^	22.52 ± 0.10^a^	59.71 ± 0.57^bc^	50.73 ± 0.43^a^	0.44
NR*	3.4–5.8	5.6–7.6	–	7.0–20.0	2.0 –8.0	45.7–80.8	56.8 –128	17.5–30.2	<1.00

Note

Values are means ± standard deviation, samples within the same column with different superscripts are significantly different (*p* ≤ .05). Keys: OFSP = Orange‐fleshed Sweet Potato; UF2 = Unfermented sorghum (56%)–soybean (17%)–OFSP (27%); F2 = Fermented sorghum (56%)–soybean (17%)–OFSP (27%); UF3 = Unfermented sorghum (59%)–soybean (31%)–OFSP (10%); F3 = Fermented sorghum (59%)–soybean (31%) ‐ OFSP (10%); CT = Cerelac; Corn starch = Basal diet.

* Normal range: Giannini et al., (1999), Diana Nicoll (2007).

Serum enzymes such as aspartate transaminase (AST), alanine transaminase (ALT), and alkaline phosphatase (ALP) are useful biomarkers of liver function (Diana, 2007). The levels of AST and ALT in serum are used in the diagnosis of health status of the liver (Kasarala & Tillmann, 2016). The AST and ALT levels in the blood are directly related to the level of tissue damage (Botros & Sikaris, 2013). The AST values of the experimental rats ranged from 15.17 to 22.52 u/L and were highest in cerelac fed group, while corn starch fed group was lowest. However, fermentation of the diet substrate significantly affects the AST response in test animals. These AST concentrations obtained were below the normal range (45.70–80.50 u/L) previously reported for healthy animals (Giannini et al., 1999; Diana, 2007). The ALT values of the experimental rats ranged from 30.43 to 50.75 u/L. The experimental rats fed with fermented samples F2 (61.33 u/L), F3 (64.30 u/L), and cerelac (59.71 u/L) had ALT values within the normal range (56.80–128.00 u/L) for healthy animals and were significantly (*p* < .05) higher than experimental rats fed with unfermented formulated diets (UF2 and UF3) and *ogi*. The ALT values were above the normal range (17.50 – 30.20 u/L) reported for healthy animals (Diana Nicoll, 2007; Giannini et al., 1999). The AST/ALT ratio ranged from 0.38 to 0.51 and was within the normal value (<1.0). This observation implies that the formulated diets are suitable for consumption and may not impair liver cell functions nor cause damage to the liver cells. A high concentration of AST or ALT in the blood is indicative of liver damage (Al‐Mamary et al., 2002; Aniagu et al., 2005).

## 4 CONCLUSION

This study revealed that locally available food commodities such as sorghum, soybean, and orange‐fleshed sweet potato can be utilized to produce a protein‐rich complementary food that is capable of combating malnutrition among children. Fermentation and soybean supplementation influenced the nitrogen retention and protein efficiency ratio in experimental animals. Fermentation increased the protein content of developed diet and had a positive impact on the nutritional indices (BV, NR, FER, PER, NPU, and TD) of the experimental animals. Fermentation can be applied to improve the protein quality of food products. Sample F3 aided growth of weanling rats and, hence, could aid growth of infants and children. A protein‐rich weaning food that is comparable to commercial weaning food (cerelac) can be strategically formulated from the blends of fermented sorghum (59%), soybean (31%), and OFSP (10%). Formulated diet (F3) can be used to manage malnutrition among infants and children as its meet the nutritional needs for infants and children. Further studies to investigate the effect of the formulated diets in clinical trials is germane.

## CONFLICT OF INTEREST

The authors declare that they do not have any conflict of interest.

## ETHICAL APPROVAL

This study was approved by the Institutional Review Board of Federal University of Technology Akure, Nigeria.

## Informed Consent

Written informed consent was obtained from all study participants.
